# Comparative Effects of Maturity and Processing on Chemical Composition and Bioactivities in *Toona sinensis* Leaves

**DOI:** 10.3390/foods14152717

**Published:** 2025-08-02

**Authors:** Guohuo Wu, Zhaoyun Chen, Yan Tang, Shuolei Xu, Wenli Fan, Li Wu, Yuntao Ji, Changqing Qu

**Affiliations:** Engineering Technology Research Center of Anti-Aging Chinese Herbal Medicine of Anhui Province, School of Biology and Food Engineering, Fuyang Normal University, Fuyang 236037, China; 15715587301@163.com (Z.C.); tangyan132@outlook.com (Y.T.); xushuoleeei@163.com (S.X.); 2023115901@stu.fynu.edu.cn (W.F.); stufynu2023115720@163.com (L.W.); jiyuntao@163.com (Y.J.)

**Keywords:** *Toona sinensis*, processing, antioxidant, hypoglycemic, metabolomic analysis

## Abstract

*Toona sinensis* (“Heiyouchun”) is a traditional Chinese woody vegetable, the leaves of which can also be processed into tea, known for its distinctive flavor and diverse bioactivities. However, the effects of leaf maturity and processing methods on its phytochemical composition and functional properties remain unclear. In this study, metabolomic analysis revealed 35 significantly different metabolites between tender and mature leaves, with higher concentrations of flavonoids, flavonoid glycosides, limonoids, and amino acids in tender leaves. Additionally, comparative analysis revealed that black tea fermentation preserves bioactive compounds more effectively than hot-air drying, particularly in tender leaves. In vitro activity assays showed that toon leaf tea extracts exhibited significant antioxidant and hypoglycemic effects, with black tea fermented tender leaves displaying the most potent bioactivity. Correlation analysis further confirmed a strong positive relationship between flavonoid/polyphenol content and bioactivity. These findings provide a theoretical foundation for optimizing processing techniques to enhance the functional properties of toon leaf tea.

## 1. Introduction

*Toona sinensis* (commonly known as Chinese toon) is an important multifunctional tree species with a long history of cultivation, a wide geographical distribution, and remarkable capacity for adaptation [1]. This species integrates food, medicinal, and material uses, showcasing its immense potential. Among its parts, the buds and tender leaves of toon are especially noteworthy. They serve as nutrient-rich vegetables, brimming with essential amino acids, vitamins, and polyunsaturated fatty acids [2]. Beyond its nutritional properties, toon has been reported to contain a wide variety of active ingredients, such as flavonoids, phenolic acids, terpenes, and alkaloids. Contemporary pharmacological research has delved deeper into the properties of toon, unveiling a wide array of bioactivities. These include antidiabetic, antioxidant, anti-inflammatory, and hepatoprotective effects [1,3].

“Heiyouchun” (*Toona sinensis*) is a well-known superior variety in China, prized for its unique flavor and nutritional profile. Its tender parts are extensively utilized in the production of vegetables, fermented sauces, and functional foods [2,4]. However, the mature toon leaves have not been fully utilized due to their poor palatability, resulting in significant resource waste. In some regions of China, mature toon leaves are commonly dried directly and used for making tea, which is believed to have auxiliary hypoglycemic effects [5]. Despite these traditional uses, the specific changes in the chemical composition and biological activities of toon leaves at different maturity stages remain unexplored, warranting further investigation.

It is well-established that processing methods can significantly influence the physicochemical properties of products, thereby affecting the recovery of bioactive components. According to Han et al., the floral characteristics associated with monoterpenoids in green tea exhibited marked differences when processed using a frying pan over fire and steam treatment [6]. Wei et al. found that the yellowing process could effectively improve the flavor quality of summer green tea [7]. In addition, Panyatip et al. reported that processing mulberry leaves using black tea techniques can elevate their flavonoid content and enhance acetylcholinesterase inhibitory activity [8]. For toon leaves, their poor palatability after direct drying has severely limited their market acceptance. However, there is a dearth of research on the effects of processing methods on the chemical composition and biological activities of toon leaves. To address this limitation, we innovatively applied black tea processing techniques to toon leaves. Given their high secondary metabolite content, we hypothesized that such processing could induce the biotransformation of bioactive compounds, thereby enhancing their functional potential. Fermenting toon leaves into tea offers dual benefits: nutritionally, it enhances nutrient accessibility and palatability; commercially, it transforms underutilized mature leaves (which are typically discarded due to poor taste) into a value-added product, reducing waste and creating market opportunities.

This study aims to systematically investigate the effects of leaf maturity and processing methodology on the phytochemical composition and bioactive properties of toon leaf tea for potential functional food applications. This research will first conduct a comparative analysis of phytochemical profiles between tender and mature toon leaves. Subsequently, toon leaf tea will be produced using two distinct processing techniques for each maturity grade. A comprehensive evaluation will then be performed to characterize processing-induced modifications in chemical constituents and assess corresponding changes in in vitro antioxidant capacity and hypoglycemic activity. The findings will provide a scientific basis for optimizing the valorization of toon resources in functional food and nutraceutical applications.

## 2. Materials and Methods

### 2.1. Chemicals

Analytical-grade solvents (acetonitrile, methanol, and formic acid) and glucose reagents were procured from Sigma-Aldrich (St. Louis, MO, USA). Chemical reference standards for rutin, quercetin, kaempferol, and gallic acid were sourced from Chem Faces (Wuhan, China). The enzymatic substrates *p*-Nitrophenyl-α-D-glucopyranoside (pNPG), α-glucosidase, and α-amylase were supplied by Yuanye Biotechnology (Shanghai, China). The ABTS⋅^+^ and DPPH⋅ detection kits were sourced from Jiancheng Bioengineering Institute (Nanjing, China). All other chemicals employed in this research met either analytical specifications or the highest purity standards commercially available.

### 2.2. Sample Preparation and UHPLC-Orbitrap-MS/MS Analysis

*T. sinensis* var. ‘Heiyouchun’ is cultivated at the Taihe County nursery (Fuyang, Anhui Province, China). In May, tender toon leaves (TTL) were collected as one bud and three to five tender leaves, while mature toon leaves (TML) were systematically harvested from the ninth node of each branch, counting upward from the shoot apex. A total of five independent replications were conducted, each consisting of leaves pooled from three individual trees. For each tree, approximately 1 kg of leaves was collected, resulting in a total of 15 kg of raw material for the study. The harvested leaves were freeze-dried and stored under vacuum at −20 °C. Sample extraction followed a previously described protocol [9] using a 70% aqueous methanol solution. For detailed analysis methods of UHPLC-Orbitrap-MS/MS, refer to “Methods S1” in the Supporting Information. TIC overlay chromatograms of QC samples are shown in Figure S1.

### 2.3. Manufacturing of Various Toon Leaf Teas

**Figure 1:**
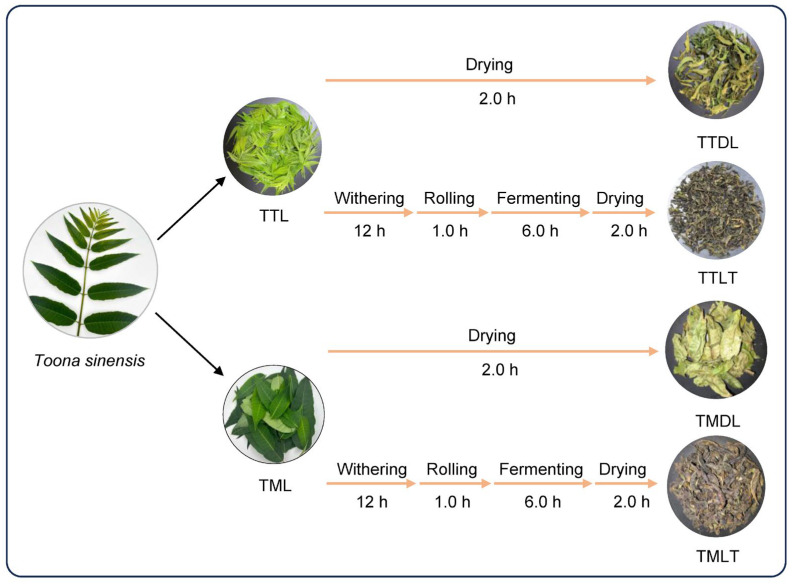
The different processing methods for toon leaf teas. TTL, toon tender leaf; TML, toon mature leaf; TTDL, toon tender dried leaves; TTLT, toon tender leaf tea; TMDL, toon mature dried leaves; TMLT, toon mature leaf tea.

The harvested toon leaves were processed via two distinct methods, as illustrated in Figure 1. For the first method, a portion of the leaves is processed into toon tender-leaf tea (TTLT) and mature-leaf tea (TMLT) using the black tea processing technique [10]. This involves a series of steps: initially, the leaves are subjected to indoor withering for a duration of 12 h. Subsequently, they are rolled for 1 h to initiate the release of essential flavors and compounds. The fermentation stage follows, which is carried out at a precisely controlled temperature of 25 ± 1 °C and a relative humidity of 85 ± 5% for 6 h. During this period, the leaves are gently turned every 2 h to ensure uniform oxidation throughout the batch. After fermentation, the leaves are dried at 110 °C for 2 h to halt further chemical reactions and preserve their quality. Finally, they are cooled for 30 min to reach a stable temperature before being labeled as TTLT and TMLT samples. The second method processes the other portion of the leaves directly. They are dried at 110 °C for 2 h and then cooled for 30 min, resulting in toon tender dried leaves (TTDL) and toon mature dried leaves (TMDL) samples. All processed samples were immediately frozen and stored at −20 °C until analysis. For homogenization, approximately 100 g of each frozen sample was finely ground using a laboratory mill and subsequently passed through a 60-mesh sieve (250 μm particle size) to ensure uniform particle distribution prior to chemical analysis.

### 2.4. Determination of Chemical Components

Chemical composition analysis was conducted utilizing well-established methods. Total flavonoids were quantified by the aluminum nitrate colorimetric method with rutin as the standard (λ = 510 nm) [11]. Total phenolic content was determined using the Folin–Ciocalteu assay, with gallic acid as the calibration standard (λ = 765 nm) [12]. Soluble sugars were measured via the anthrone-sulfuric acid assay (glucose standard, λ = 620 nm) [13], while soluble proteins were assessed using the Bradford assay (BSA standard, λ = 595 nm). Free amino acids were analyzed using the ninhydrin reaction (glycine standard, λ = 570 nm). All spectrophotometric measurements were performed in triplicate using a Shimadzu UV2600 spectrophotometer, with appropriate blank corrections. Detailed calibration curve parameters are provided in Table S1. Chemical component contents were expressed as mg/g of dry matter of toon leaf tea.

### 2.5. Quantification of Rutin, Quercetin, and Kaempferol Using UPLC

Based on previous reports [14] and our optimization experiments, extractions were performed under the following optimized conditions: a 100 mg sample of toon leaf tea powder underwent two successive 30 min extractions at 60 °C in a water bath, using 8.0 mL of a formic acid/methanol solution (5:95, *v*/*v*) each time. After centrifugation at 3500 rpm for 10 min, the combined supernatants were diluted to a final volume of 10.0 mL with the same formic acid/methanol mixture. The diluted supernatant was subsequently filtered through a 0.22 μm membrane filter to prepare it for UPLC analysis as previously described [15]. For detailed analysis parameters, refer to “Methods S2” in the Supporting Information. The UPLC calibration curves exhibited excellent linearity (R^2^ > 0.998) across the tested concentration ranges for all standards, as outlined in Table S2.

### 2.6. Measurement of α-Glucosidase and α-Amylase Inhibitory Activity

Inhibition assays for α-glucosidase and α-amylase were conducted following established protocols [16], with slight modifications. Prior to inhibition assays, to prepare the tea water extract, 50 g of tea powder was weighed and boiled in 1000 mL ultrapure water with constant stirring at 85 °C for 30 min. Then, ultrasonic extraction was conducted at 75 °C for 30 min (100 W power). The resulting solution was then filtered, concentrated, and freeze-dried to obtain the final extract. For the α-glucosidase inhibition assessment, a reaction mixture comprising 20 μL of various toon leaf tea water extracts (or 1 mM acarbose as a positive control) and 20 μL of an enzyme solution (1 U/mL in PBS, pH 6.8) was incubated at 37 °C. After adding pNPG and subsequent incubation at the same temperature, the absorbance was measured at 405 nm. The IC_50_ value was derived using non-linear regression, based on the logarithmic correlation between the inhibition percentage and the concentration of toon leaf tea water extract. For the assessment of α-amylase inhibition, a reaction mixture was prepared by combining 80 μL of various toon leaf tea water extracts (or 1 mM acarbose as a positive control) with 80 μL of an enzyme solution (1 U/mL in PBS, pH 6.8). This mixture was incubated at 37 °C. Subsequently, 40 μL of a 1% soluble starch solution in PBS was added, and the incubation continued at 37 °C for an additional 10 min. After the addition of a DNS reagent and heating at 100 °C for 10 min, the absorbance of the resulting solution was measured at 540 nm. The α-amylase activity was then calculated following the previously established methodology [17].

### 2.7. Determination of Antioxidant Activity

The radical scavenging activities of ABTS⋅^+^ and DPPH⋅ in various toon leaf teas were evaluated using commercial reagent kits from Jiancheng Bioengineering Institute. For the ABTS⋅^+^ radical scavenging assay, 20 μL of various toon leaf tea water extracts at different concentrations were mixed with 180 μL of ABTS⋅^+^ solution and incubated at 25 °C for 6 min. Subsequently, the absorbance was measured at 405 nm. The percentage inhibition was determined using the formula: % Inhibition = (A_0_ − A_1_)/(A_0_ − A_2_) × 100, where A_0_ represents the absorbance of 180 μL ABTS⋅^+^ solution alone in methanol, A_1_ is the absorbance of 80 μL ABTS⋅^+^ solution mixed with the sample in methanol, and A_2_ denotes the absorbance of the methanol solvent alone. Regarding the DPPH⋅ radical scavenging assay, 20 μL of samples at varying concentrations were combined with 90 μL of DPPH⋅ solution and incubated at 25 °C for 30 min. The absorbance was then measured at 517 nm, and the percentage inhibition was calculated using the same formula as described for the ABTS⋅^+^ assay.

### 2.8. Statistical Analysis

A statistical analysis was conducted using Prism 8.0 (GraphPad Software, San Diego, CA, USA). Differences between multiple groups or between two groups were evaluated using one-way ANOVA or *t* tests, with a significance level of 0.05. Results are presented as means ± SEM.

## 3. Results

### 3.1. Analysis of the Metabolite Profiles of Tender and Mature Toon Leaves

**Figure 2:**
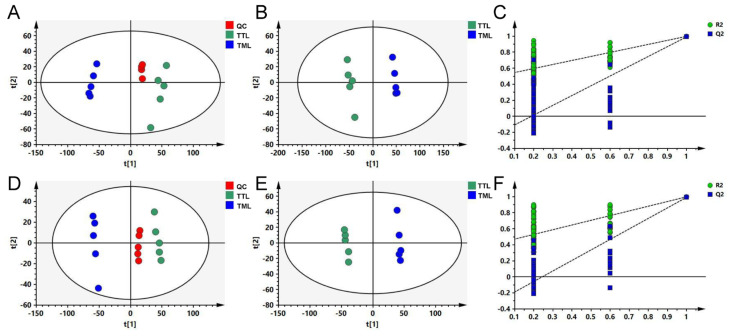
Multivariate statistical analysis between TTL and TML. (**A**,**D**) depict the PCA score plots for positive (R^2^X = 0.902; Q^2^ = 0.771) and negative (R^2^X = 0.911; Q^2^ = 0.780) ionization modes, respectively. (**B**,**E**) showcase the PLS-DA score plots for positive (R^2^X = 0.842; R^2^Y = 0.998; Q^2^ = 0.992) and negative (R^2^X = 0.888; R^2^Y = 0.999; Q^2^ = 0.993) ionization modes, correspondingly. (**C**,**F**) present the PLS-DA model validation results for positive (R^2^ = 0.497; Q^2^ = −0.228) and negative (R^2^ = 0.413; Q^2^ = −0.319) ionization modes, respectively.

To investigate the effects of toon leaf maturity on its chemical composition, two different maturity levels of leaves (TTL and TML) were analyzed using UHPLC-Orbitrap-MS/MS in this study. As has been previously documented, an untargeted metabolomics approach holds significant potential for comprehensively profiling secondary metabolites in plants [18]. By scrutinizing the typical total ion current chromatograms of the samples, we identified 3641 ion features in positive ion mode and 2725 in negative ion mode. Initially, an unsupervised PCA was conducted to assess the differences between TTL and TML. In the positive ion mode, PCA revealed that TTL samples clustered in the first and fourth quadrants, whereas TML samples were grouped in the second and third quadrants (Figure 2). A comparable pattern emerged from the negative ion mode data (Figure 2). Furthermore, the QC samples clustered closely, underscoring the stability and reliability of our metabolomics analysis. These results suggest that TTL and TML possess distinct chemical profiles. PLS-DA has previously been shown to perform better than PCA to determine variation within a group [19]. To further evaluate the differences in chemical profiles between TTL and TML, supervised PLS-DA statistical analysis was employed. The results showed that TTL samples clustered in quadrants II and III, while TML samples were grouped in quadrants I and IV (Figure 2). This finding reinforced the notion that TTL and TML exhibit distinct chemical compositions. The model parameters, including R^2^X, R^2^Y, and Q^2^, were calculated as 0.842, 0.998, and 0.992 for the positive ion mode, and 0.888, 0.999, and 0.993 for the negative ion mode, respectively. These values indicate successful model establishment without overfitting. A permutation test further confirmed the absence of overfitting in both ion modes (Figure 2).

**Figure 3:**
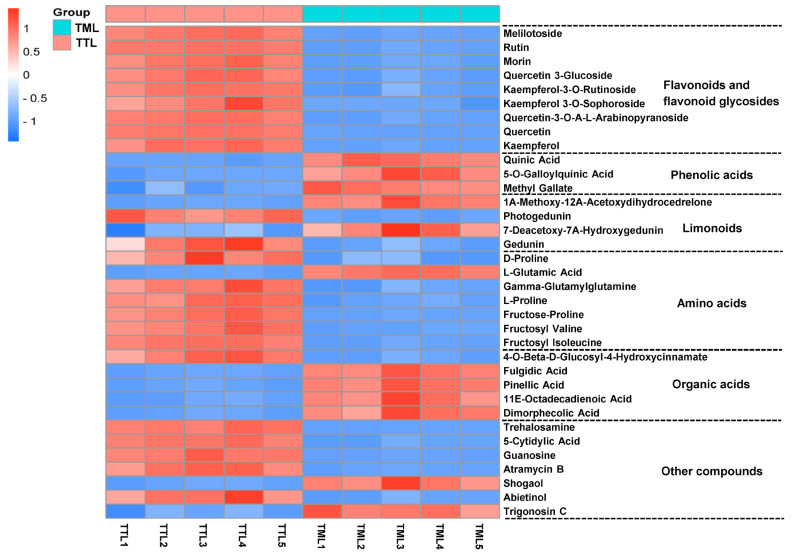
Heat map of the metabolite content of TTL and TML. Red boxes denote values exceeding the mean, while blue boxes indicate values below it.

According to the previous method [20], a screening criterion was set up to pinpoint potential differential metabolites, which necessitated a VIP value exceeding 1 (VIP > 1), a *p*-value from the nonparametric test below 0.05 (*p* < 0.05), and a fold change of either greater than 2.0 or less than 0.5 (to cover both upregulation and downregulation). By cross-referencing retention time (RT), *m*/*z* values, and secondary mass spectrometry fragments, and by consulting multiple metabolome databases alongside the existing literature, we successfully identified a total of 35 differential metabolites between TTL and TML. These compounds encompassed nine flavonoids and flavonoid glycosides, three phenolic acids, four limonoids, seven amino acids, five organic acids, and seven additional compounds (Table 1). Utilizing the abundance data of these 35 differential metabolites, a heat map was generated to visually depict the disparities in metabolite abundance between TTL and TML (Figure 3).

#### 3.1.1. Flavonoids and Flavonoid Glycosides

Flavonoids and flavonoid glycosides are a group of compounds that are well-known for their bioactive properties [21]. It has been reported that toon leaves are rich in flavonoids [1]. Our comparative analysis revealed that the contents of nine flavonoids and flavonoid glycosides were found to differ between TTL and TML, as detailed in Table 1 and Figure 3. These metabolites were identified as melilotoside, rutin, morin, quercetin 3-glucoside, kaempferol-3-O-rutinoside, kaempferol 3-O-sophoroside, quercetin-3-O-A-L-arabinopyranoside, quercetin, and kaempferol. Compared to TML, all of these compounds exhibited higher levels in TTL. The most pronounced differences were observed for rutin (3.88-fold higher in TTL), quercetin (11.64-fold higher), and kaempferol (4.6-fold higher). The higher glycosylated flavonoid content in TTL suggests greater metabolic activity in young leaves, as glycosylation often represents an active storage or transport form of these compounds. A previous study reported that tender lotus leaves contain more quercetin, rutin, and total flavonoids than mature leaves, which is consistent with our findings [22].

#### 3.1.2. Phenolic Acids

Toon leaves are rich in phenolic acids. Numerous studies have demonstrated that phenolic acids, serving as crucial astringent compounds, play a pivotal role in influencing the taste profile of leaves [23]. In this study, phenolic acids, including quinic acid, 5-O-galloylquinic acid, and methyl gallate, were screened and showed significant differences between TTL and TML, as illustrated in the heat map (Figure 3). Specifically, quinic acid, 5-O-galloylquinic acid, and methyl gallate exhibited lower contents in TTL than in TML. Li et al. demonstrated that the quinic acid content in Yuexi Cuilan tea increased with leaf maturity, with the youngest leaves containing the lowest levels [24], a finding consistent with our own research. The lower phenolic acid levels in TTL could have important implications for their sensory properties and potential applications. Since phenolic acids contribute to astringency, their reduced presence in tender leaves may result in a milder taste, making TTL more palatable for culinary uses.

#### 3.1.3. Limonoids

Limonoids, a distinctive class of tetracyclic triterpenoid derivatives featuring a characteristic furanolactone scaffold, represent major bioactive compounds in *T. sinensis* [1]. Recent studies highlight their diverse pharmacological potential. Fu et al. first reported the presence of these phytochemicals in fresh young leaves and shoots of toon, along with their neuroprotective properties [25]. Further research by Hu et al. elucidated the multifaceted bioactivities of toon-derived limonoids, including potent antioxidant effects via free radical scavenging, anti-inflammatory action mediated by NF-κB pathway suppression, and selective cytotoxicity toward HepG2 hepatocellular carcinoma cells [26]. The metabolic data revealed that four limonoids differed between TTL and TML (Figure 3). TTL showed lower levels of 1A-methoxy-12A-acetoxydihydrocedrelone and 7-deacetoxy-7A-hydroxygedunin than TML. However, TTL contained significantly higher levels of limonoids (photogedunin and gedunin) than TML. These compounds have demonstrated notable bioactivities in previous studies. For instance, research has shown that gedunin and photogedunin from *Xylocarpus granatum* exhibit antifilarial activity against *Brugia malayi* and protective effects against peptic ulcers in rats [27,28]. The elevated concentrations of these bioactive compounds in TTL suggest that toon tender leaves may have greater potential for disease prevention and treatment applications.

#### 3.1.4. Amino Acids and Organic Acids

Amino acids are crucial for the umami taste and aroma [29]. Our findings demonstrated that two different maturity levels of leaves exhibited differences in the levels of seven amino acids (Figure 3). Notably, six amino acids—D-proline, gamma-glutamylglutamine, L-proline, fructose-proline, fructosyl valine, and fructosyl isoleucine—showed significantly higher concentrations in TTL, while L-glutamic acid exhibited an inverse pattern, with greater abundance in TML. This differential accumulation pattern suggests distinct metabolic priorities at different developmental stages: tender leaves may preferentially accumulate certain amino acids to support rapid growth and protein synthesis, while mature leaves invest more in glutamate metabolism, potentially for nitrogen storage or stress responses.

Organic acids are acidic organic compounds comprising carboxyl groups and are widely present in plants [30]. They participate in the metabolism of plants, regulate nutrient absorption and growth, and contribute to aroma and taste quality. Five differential organic acids were identified between TTL and TML: 4-O-beta-D-glucosyl-4-hydroxycinnamate, fulgidic acid, pinellic acid, 11E-octadecadienoic acid, and dimorphecolic acid (Figure 3). TTL exhibited relatively lower levels of fulgidic acid, pinellic acid, 11E-octadecadienoic acid, and dimorphecolic acid compared to TML. These organic acids can have diverse effects on the sensory properties of the leaves. Fulgidic acid and pinellic acid may contribute to the overall acidity and astringency of the leaves, while 11E-octadecadienoic acid and dimorphecolic acid could be involved in the formation of volatile compounds that contribute to the aroma. The lower levels of these organic acids in TTL may lead to a less acidic and astringent taste and a potentially different aroma profile compared to TML. In contrast, the level of 4-O-beta-D-glucosyl-4-hydroxycinnamate, a single metabolite, was higher in TTL than in TML. Overall, the differences in amino acid and organic acid levels between TML and TTL highlight the importance of leaf maturity in determining the chemical composition and sensory properties of toon leaves. These findings have significant implications for the utilization of toon leaves.

#### 3.1.5. Other Compounds

The metabolic analysis also revealed seven differential metabolites of other compounds between TTL and TML, including trehalosamine, 5-cytidylic acid, guanosine, atramycin B, shogaol, abietinol, and trigonosin C (Figure 3). Trehalosamine, 5-cytidylic acid, guanosine, atramycin B, and abietinol levels were higher in TTL than in TML. In contrast, shogaol and trigonosin C showed lower levels in TTL than in TML.

### 3.2. Quantification of Active Components in Various Toon Leaf Teas

**Figure 4:**
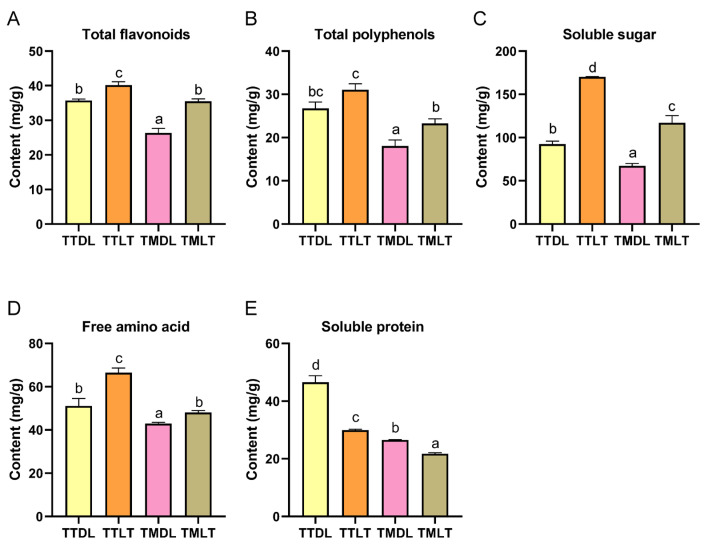
Quantitative comparison of 5 bioactive compounds in various toon leaf teas. (**A**) Total flavonoid levels; (**B**) total polyphenol levels; (**C**) soluble sugar levels; (**D**) free amino acid levels; and (**E**) soluble protein levels. Values are means and SEM (n = 3). Columns with different letters are significantly different (*p* < 0.05).

Next, fresh toon leaves at varying maturity stages were processed using two distinct methods, and the active components in the resulting teas were quantified. Previous UHPLC-Orbitrap-MS/MS analyses had already revealed metabolite differences between tender and mature toon leaves at the raw material stage, and this study further examined how processing methods influence and amplify these variations. As shown in Figure 4, the total flavonoids of TTDL and TTLT were 35.68 and 40.17 mg/g of dry weight (DW), respectively, which were higher than those of TMDL (26.31 mg/g) and TMLT (35.48 mg/g). Similarly, the total polyphenols of TTDL (26.74 mg/g) and TTLT (31.05 mg/g) were higher than those of TMDL (18.03 mg) and TMLT (23.26 mg/g). In this study, the total flavonoid and polyphenol contents varied among different groups, with tender leaves exhibiting higher levels compared to mature leaves, aligning with prior UHPLC-Orbitrap-MS/MS findings. Notably, Ma et al.’s research observed analogous variations in total flavonoids and polyphenols across different maturity stages in fresh lotus leaf extracts [22]. Furthermore, the total flavonoid and polyphenol contents in the black tea processed groups were consistently higher than in the hot-air-dried groups, regardless of whether the leaves were tender or mature. Traditionally, black tea fermentation has been perceived as predominantly an enzymatic oxidation process, independent of microbial involvement. However, recent studies have suggested the potential presence of microbial activity during black tea processing, which could influence the chemical composition and enhance the formation of specific flavor compounds [31]. While these findings shed light on black tea fermentation, the specific microbial role in toon leaf tea fermentation remains unexplored. To address this knowledge gap, comprehensive studies incorporating microbiological analyses are needed to investigate the potential synergistic effects between enzymatic oxidation and microbial fermentation in processed toon leaves. Based on the current evidence, we propose that the black tea processing of toon leaves involves a synergistic mechanism, which may enhance the flavonoid and polyphenol content in the final product.

Soluble sugars, free amino acids, and proteins are crucial nutritional constituents of toon leaves. Quantitative assessments of soluble sugars and free amino acids across diverse toon leaf teas revealed that tender leaves generally contained higher levels of these components compared to mature leaves (TTDL > TMDL; TTLT > TMLT). Furthermore, black tea processed groups exhibited elevated levels of soluble sugars and free amino acids relative to hot-air-dried processed groups (TTLT > TTDL; TMLT > TMDL). However, an inverse trend was observed for soluble protein content, with black tea processed groups displaying lower levels than their hot-air-dried counterparts (TTLT < TTDL; TMLT < TMDL). Amino acids and their derivatives, serving as pivotal taste-active metabolites in tea infusions, play a fundamental role in regulating the sensory attributes of tea extracts and are instrumental in shaping the intricate flavor profile of tea. This study demonstrated that while black tea processing techniques significantly diminish the soluble protein content in toon leaves, they concurrently enhance the total amino acid content. This compositional shift contributes to the enhanced flavor expression of toon leaf tea. Previous studies have shown that the withering process facilitates the hydrolysis of proteins into free amino acids [32]. Similarly, our study reveals that the withering phase in black tea production significantly elevates the free amino acid content in toon leaves. This discovery not only deepens our comprehension of the mechanisms underlying flavor compound transformation during tea processing but also establishes a scientific basis for modulating the flavor profile of toon leaf tea, thereby offering valuable insights for optimizing the production and quality of toon leaf tea.

**Figure 5:**
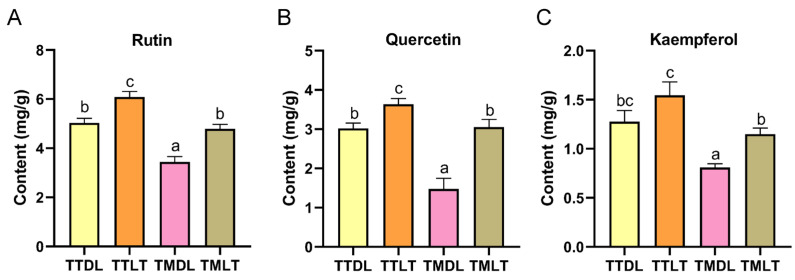
Quantification of rutin, quercetin, and kaempferol in various toon leaf teas. (**A**) Rutin content, (**B**) quercetin content, and (**C**) kaempferol content. Values are means and SEM (n = 3). Columns with different letters are significantly different (*p* < 0.05).

An untargeted metabolomics analysis unveiled a notable disparity in the flavonoid and flavonoid glycoside contents between toon tender leaves and their mature counterparts, with the former exhibiting significantly higher relative amounts. To substantiate these observations, we proceeded with an absolute quantitative analysis of various toon leaf tea samples. UPLC was employed to quantify rutin, quercetin, and kaempferol using their respective standard chemicals. The quantitative results demonstrated that toon tender leaves had higher levels of rutin, quercetin, and kaempferol than toon mature leaves (Figure 5; TTDL > TMDL; TTLT > TMLT), which aligns with the chemical profile data. In addition, our results showed that rutin, quercetin, and kaempferol in black-tea-processed groups were higher than in hot-air-dried processing groups (TTLT > TTDL; TMLT > TMDL). This trend aligns precisely with the total flavonoid content patterns observed, further underscoring the impact of processing methods on flavonoid composition.

Consequently, both processing methods and raw material tenderness significantly influence the chemical composition of toon leaf tea. Clarifying these relationships offers a scientific basis for refining processing techniques to produce higher-quality toon leaf tea products.

### 3.3. Antioxidant Capacity Determination of Various Toon Leaf Teas

**Figure 6:**
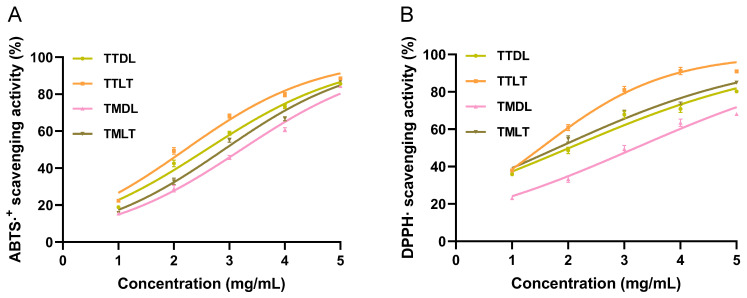
Scavenging abilities of various toon leaf teas regarding ABTS⋅^+^ and DPPH. (**A**) ABTS⋅^+^ scavenging ability; (**B**) DPPH scavenging ability. Columns with different letters are significantly different (*p* < 0.05).

ABTS⋅^+^ and DPPH⋅ radical scavenging assays are well-established and widely employed antioxidant methods, serving as indicators of a sample’s capacity to donate hydrogen atoms [33]. In this study, we assessed the ABTS⋅^+^ and DPPH⋅ radical scavenging abilities of four different toon leaf tea extracts. The ABTS⋅^+^ radical scavenging abilities of the four toon leaf tea extracts are presented in Figure 6A. It was observed that the ABTS⋅^+^ radical scavenging rates of these extracts exhibited a steady increase as the concentration rose from 1 mg/mL to 5 mg/mL. The IC_50_ values, which represent the concentration required to achieve 50% radical scavenging, for TTDL, TTLT, TMDL, and TMLT were determined to be 2.32, 1.98, 2.99, and 2.67 mg/mL, respectively (Table S3). Regarding the DPPH⋅ radical scavenging activity, as depicted in Figure 6B, the four toon leaf tea extracts displayed varying degrees of efficacy, following the order TTLT > TMLT > TTDL > TMDL. Our results unequivocally demonstrate that all the various toon leaf tea extracts possess positive scavenging capacities against both ABTS⋅^+^ and DPPH⋅ radicals (Table S4). Tender leaf tea exhibited a more pronounced free radical scavenging ability compared to mature leaf tea. Moreover, the black-tea-processed toon leaf tea exhibits a superior scavenging capacity compared to the hot-air-dried processed toon leaf tea.

### 3.4. Comparison of α-Glucosidase and α-Amylase Inhibitory Activity in Various Toon Leaf Teas

Currently, α-glucosidase and α-amylase inhibitors are available for clinical use, including Acarbose and Voglibose, for managing blood glucose levels in patients with T2DM [34]. Nevertheless, their synthetic nature often leads to various gastrointestinal adverse effects, such as abdominal distension, pain, and diarrhea [35]. Therefore, the quest to discover natural, non-toxic α-glucosidase inhibitors from the environment has increasingly garnered attention. Our in vitro experiments demonstrated that water extracts of four various toon leaf teas inhibited the activity of α-glucosidase and α-amylase. As shown in Table 2, the α-glucosidase IC_50_ values for TTDL, TTLT, TMDL, and TMLT were 743.40, 471.60, 1110.00, and 875.30 μg/mL, respectively. The inhibitory activity against α-glucosidase followed the order: TTLT > TTDL > TMLT > TMDL. Furthermore, the α-amylase inhibitory effects of toon leaf tea extracts displayed a comparable trend to that of α-glucosidase inhibition. This suggests that toon leaf tea may possess a potential synergistic mechanism in modulating carbohydrate-digesting enzymes.

### 3.5. Correlation Analysis Between Active Components and Bioactivities in Various Toon Leaf Teas

**Figure 7:**
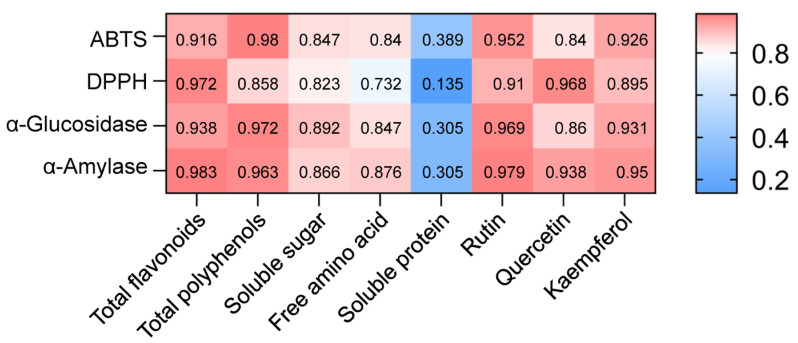
Correlation heat map between active components and bioactivities in various toon leaf teas.

As illustrated in Figure 7, the Pearson test revealed positive correlations among the active components and bioactivities of various toon leaf teas. Notably, the strongest correlation was observed between α-amylase inhibitory activity and total flavonoid content, with a correlation coefficient (R^2^) of 0.983. Furthermore, DPPH⋅ radical scavenging capacity exhibited a robust correlation with total flavonoid content (R^2^ = 0.972), a finding consistent with previous reports on lotus leaves [22]. Similarly, ABTS⋅^+^ radical scavenging capacity and α-glucosidase inhibitory activity were strongly correlated with total polyphenol content, with R^2^ values of 0.98 and 0.972, respectively. The health-promoting potential of phenolic compounds is largely attributed to their antioxidant properties, which involve the donation of a hydrogen atom from the aromatic hydroxyl group to neutralize free radicals [36]. Additionally, ABTS⋅^+^ and DPPH⋅ radical scavenging capacities, along with α-glucosidase and α-amylase inhibitory activities, demonstrated strong correlations with rutin content (each with R^2^ > 0.9). In contrast, the correlations between these bioactivities and soluble sugar, free amino acid, and soluble protein contents in toon leaf were relatively weak, particularly with soluble proteins (each with R^2^ < 0.4). While these statistical associations suggest potential relationships between flavonoid/phenolic compound levels and bioactivities in toon leaf tea extracts, we acknowledge that further mechanistic studies would be required to establish causal relationships.

## 4. Conclusions

In this study, the effects of maturity and processing on the chemical composition and bioactivities of toon leaves were investigated. A metabolomic analysis revealed that the two leaf maturity stages induced significant changes in 35 metabolites, including flavonoids, flavonoid glycosides, limonoids, amino acids, etc. Toon leaves at two maturity stages were processed into tea via hot air drying or black tea fermentation. Our findings reveal that both the leaf maturity stage and processing methods exert an influence on the phytochemical composition, as well as the antioxidant and hypoglycemic properties, of toon leaf tea. Toon leaves at the tender stage or those treated with the black tea process had more active components, including flavonoids, polyphenols, soluble sugar, and free amino acids. For example, black-tea-fermented tender leaves had 52.69% more flavonoids and 72.15% more polyphenols than hot-air-dried mature leaves. Further testing of the water extracts from various toon leaf teas revealed good antioxidant and in vitro hypoglycemic activities. Using the DPPH radical scavenging assay, we found that the IC_50_ value of the water extract from black-tea-fermented tender leaves was 1.39 mg/mL, significantly lower than that of other samples (1.39–2.91 mg/mL). Similarly, the α-glucosidase inhibition assay revealed that the IC_50_ value for the in vitro hypoglycemic activity of this extract was 0.47 mg/mL, the lowest among all tested samples. A significant positive correlation was observed between these activities and the contents of flavonoids and polyphenols. In summary, our study highlights two key findings. Firstly, leaf maturity substantially influences the chemical composition of toon leaves, with tender leaves containing higher levels of active components. Secondly, black tea fermentation outperforms hot air drying in enhancing the active component content and biological activities of toon leaf tea. These results suggest that black tea fermented tender toon leaves hold promise for producing teas with superior antioxidant and hypoglycemic properties.

However, it should be noted that our study has some limitations. The activity assessments were only conducted in vitro, and further in vivo studies are required to validate the biological effects observed. Additionally, the long-term stability and safety of these toon leaf teas need to be evaluated. Future research should focus on in vivo experiments to confirm the health benefits, optimize the processing parameters to further improve the quality of the teas, and explore the potential industrial applications of these innovative natural health products.

## Figures and Tables

**Table 1:** The differentiated metabolites of TTL and TML.

Compound Name	RT (min)	Adduct *m*/*z*	Formula	Ion Mode	Fragments	VIP	*p*-Value	FC(TTL/TML)
**Flavonoids and** **flavonoid glycosides**								
Melilotoside	9.44	325.0926	C15H18O8	[M − H]−	163, 119, 93	1.36	7.00 × 10^−11^	3.13
Rutin	11.51	609.146	C27H30O16	[M − H]−	609, 301, 300, 271, 151	3.39	1.00 × 10^−12^	3.88
Morin	11.86	303.0497	C15H10O7	[M + H]+	303, 257, 229, 153, 137	5.25	1.00 × 10^−9^	3.27
Quercetin 3-Glucoside	11.86	465.1028	C21H20O12	[M + H]+	303, 229, 153, 85	9.13	3.00 × 10^−9^	3.13
Kaempferol-3-O-Rutinoside	12.13	593.1503	C27H30O15	[M − H]−	593, 285, 255, 227	1.39	4.00 × 10^−9^	2.12
Kaempferol 3-O-Sophoroside	12.24	611.1611	C27H30O16	[M + H]+	465, 303, 145, 85	1.11	8.00 × 10^−8^	3.12
Quercetin-3-O-A-L-Arabinopyranoside	12.30	433.0773	C20H18O11	[M − H]−	433, 300, 271, 255	3.32	3.00 × 10^−11^	3.72
Quercetin	14.65	301.0352	C15H10O7	[M − H]−	301, 178, 151, 107	1.65	3.00 × 10^−13^	11.64
Kaempferol	15.74	285.0404	C15H10O6	[M − H]−	285, 151, 137, 93	1.23	7.00 × 10^−10^	4.60
**Phenolic acids**								
Quinic Acid	1.38	193.0708	C7H12O6	[M + H]+	178, 133, 122	1.52	8.00 × 10^−9^	0.38
5-O-Galloylquinic Acid	4.43	345.0819	C14H16O10	[M + H]+	153, 125	1.11	1.00 × 10^−7^	0.18
Methyl Gallate	9.38	183.03	C8H8O5	[M − H]−	183, 124, 78	1.45	1.00 × 10^−7^	0.41
**Limonoids**								
1A-Methoxy-12A-Acetoxydihydrocedrelone	17.20	307.1914	C18H28O4	[M − H]−	189, 235, 185, 121	1.05	1.00 × 10^−8^	0.28
Photogedunin	17.53	513.2123	C28H34O9	[M − H]−	513, 495	2.64	8.00 × 10^−9^	3.99
7-Deacetoxy-7A-Hydroxygedunin	20.04	441.2274	C26H32O6	[M + H]+	441 423, 423, 161	1.47	1.00 × 10^−5^	0.54
Gedunin	20.57	483.2376	C28H34O7	[M + H]+	423, 379, 161, 137	2.09	1.00 × 10^−5^	3.07
**Amino acids**								
D-Proline	1.60	116.0706	C5H9NO2	[M + H]+	70	1.03	3.00 × 10^−6^	2.21
L-Glutamic Acid	1.28	148.0604	C5H9NO4	[M + H]+	129, 84	3.64	1.00 × 10^−10^	0.29
Gamma-Glutamylglutamine	1.36	276.1191	C10H17N3O6	[M + H]+	147, 130	4.10	8.00 × 10^−8^	2.34
L-Proline	1.37	116.0706	C5H9NO2	[M + H]+	116, 70, 43	2.93	1.00 × 10^−8^	2.86
Fructose-Proline	1.41	278.1234	C11H19NO7	[M + H]+	260, 242, 214, 128	6.52	1.00 × 10^−9^	7.31
Fructosyl Valine	1.47	280.1391	C11H21NO7	[M + H]+	244, 216, 198, 118	3.34	5.00 × 10^−9^	2.81
Fructosyl Isoleucine	2.45	292.1401	C12H23NO7	[M − H]−	130, 101	1.36	7.00 × 10^−11^	3.57
**Organic acids**								
4-O-Beta-D-Glucosyl-4-Hydroxycinnamate	9.44	344.134	C15H17O8	[M + NH4]+	165, 147, 119	2.74	1.00 × 10^−7^	2.93
Fulgidic Acid	15.31	327.2175	C18H32O5	[M − H]−	327, 229, 171	2.75	2.00 × 10^−9^	0.33
Pinellic Acid	15.88	329.2334	C18H34O5	[M − H]−	329, 229, 211, 171	1.85	3.00 × 10^−9^	0.36
11E-Octadecadienoic Acid	20.20	293.2121	C18H30O3	[M − H]−	275, 223, 195	3.39	1.00 × 10^−7^	0.43
Dimorphecolic Acid	20.92	295.2277	C18H32O3	[M − H]−	277, 195, 171	2.48	9.00 × 10^−8^	0.44
**Other compounds**								
Trehalosamine	1.21	342.1395	C12H23NO10	[M + H]+	324, 306, 288, 174	4.99	4.00 × 10^−11^	4.60
5-Cytidylic Acid	1.41	324.1294	C9H14N3O8P	[M + H]+	324, 112	1.53	3.00 × 10^−11^	3.37
Guanosine	3.70	282.0843	C10H13N5O5	[M + H]+	150, 133, 108	1.62	2.00 × 10^−11^	5.61
Atramycin B	8.44	451.1391	C25H24O8	[M − H]−	361, 289, 171, 128	1.15	1.00 × 10^−7^	6.98
Shogaol	20.05	277.2159	C17H24O3	[M + H]+	135, 93, 91, 79	7.99	1.00 × 10^−7^	0.33
Abietinol	22.08	289.2527	C20H32O	[M + H]+	271, 201, 149, 121, 107, 93	2.83	1.00 × 10^−7^	3.11
Trigonosin C	24.14	609.2702	C34H40O10	[M + H]+	609, 591	4.05	1.00 × 10^−7^	0.46

Note: Values are means SE (n = 5). TTL, toon tender leaf; TML, toon mature leaf; RT, retention time; VIP, variable importance in projection; FC, fold change.

**Table 2:** IC_50_ values of toon leaf for α-glucosidase and α-amylase inhibitory activities.

Sample	α-Glucosidase IC_50_ (μg/mL)	α-Amylase IC_50_ (μg/mL)
TTDL	743.40 ± 27.94 ^b^	1911.00 ± 42.61 ^b^
TTLT	471.60 ± 28.08 ^a^	1034.00 ± 82.48 ^a^
TMDL	1110.00 ± 23.66 ^d^	3943.00 ± 75.10 ^d^
TMLT	875.30 ± 27.61 ^c^	2312.00 ± 41.20 ^c^

Note: Different letters within columns denote statistically significant differences between groups (*p* < 0.05).

## Data Availability

The original contributions presented in this study are included in the article/supplementary material. Further inquiries can be directed to the corresponding author.

## Supplementary Materials

The following supporting information can be downloaded at: https://www.mdpi.com/article/10.3390/foods14152717/s1, Figure S1: TIC overlay chromatograms of QC samples in positive and negative ion modes; Table S1: Linear equations and correlation coefficients (R^2^) of five bioactive compounds; Table S2: Linear equations and correlation coefficients (R^2^) of rutin, quercetin, and kaempferol; Table S3: IC_50_ values of toon leaf for ABTS⋅^+^ and DPPH⋅ radical scavenging activities; Table S4: Antioxidant activities of toon leaf tea extracts determined using DPPH assays; Method S1: UHPLC-Orbitrap-MS/MS analysis; Method S2: UPLC analysis method.
