# Supplementary material for: Comparative Effects of Maturity and Processing on Chemical Composition and Bioactivities in Toona sinensis Leaves
Source: Foods. 2025 Aug 2;14(15):2717. doi: 10.3390/foods14152717 (PMC12346186; doi:10.3390/foods14152717)
Supplement: Supplementary file 1 [file foods-14-02717-s001.zip › foods-3757404-supplementary.pdf]

Supplement Information

Method S1: UHPLC-Orbitrap-MS/MS analysis

The sample was extracted with 70% aqueous methanol and sonicated for 30 minutes. Following that, 2 mL of the supernatant were collected, and the methanol was completely evaporated using nitrogen gas. The dried residue was then dissolved in 2 mL of pure water and loaded onto an activated C18 extraction column. The column was rinsed with 1 mL of pure water, followed by a wash with 2 mL of pure methanol. After centrifugation at 12,000 r/min for 10 minutes, 400 μL of the supernatant were collected. This supernatant was dried completely using nitrogen gas, and the residue was dissolved in 400 μL of 70% methanol. The solution was centrifuged again at 12,000 r/min for 10 minutes, and the resulting supernatant was harvested for further analysis. Quality control (QC) samples were prepared by mixing equal volumes of each sample to monitor system stability during untargeted metabolomics analysis.

The raw UHPLC-Orbitrap-MS/MS data were processed through multivariate statistical analysis using XCMS Online. Principal Component Analysis (PCA) and Partial Least Squares-Discriminant Analysis (PLS-DA) were executed in SIMCA-P 13.0 (Umetrics, Sweden). To ensure model validity and mitigate overfitting risks, permutation testing involving 200 iterative validations was implemented. Critical model parameters were derived from the PLS-DA framework, encompassing Variable Importance in Projection (VIP) scores, fold-change (FC = TTL/TML) ratios, and statistical probability values (p-values). Metabolite differentiation between TTL and TML groups was established through multi-criteria screening: relative standard deviation (RSD) < 30%, VIP scores exceeding 1.0, statistical significance threshold ($p < 0.05$), and FC thresholds set at $|\log2(FC)| > 1.0$ (equivalent to FC > 2.0 or < 0.5) (Li et al., 2021). Candidate metabolites were provisionally annotated through chromatographic (retention time) and spectrometric (m/z) matching against established databases (HMDB, http://www.hmdb.ca/) and PubChem (https://pubchem.ncbi.nlm.nih.gov/), supplemented by literature verification. Final metabolic pattern visualization was achieved through hierarchical clustering analysis using MetaboAnalyst 5.0 (https://www.metaboanalyst.ca/), presented as a colorimetric heatmap.

Method S2 UPLC analysis method

Chromatographic separation was achieved using an ACQUITY UPLC HSS T3 column (150 mm × 2.1 mm, 1.8 μm, Thermo Scientific), utilizing a mobile phase composed of an acetonitrile/water (5:95, v/v) solution (eluent A) and water-0.1% formic acid/acetonitrile (5:95, v/v) solution (eluent B). For analysis, a 2 μL sample volume was injected into the column at a temperature of 30 °C. The gradient program was set as follows: mobile phase B from 0 to 2% (v/v) was initiated in 1 min, from 2 to 15% (v/v) in 1 min, from 15 to 26% (v/v) in 6 min, from 26 to 100% (v/v) in 1 min, maintained 1 min, then from 100 to 2% (v/v) in 1 min, final equilibration time of 1 min at 2% (v/v) B at a flow rate of 0.4 mL/min for the linear gradient. The sample was detected at a wavelength of 254 nm.

Table S1 Linear equations and correlation coefficients (R^2) of five bioactive compounds.

Compound	Reference standard	Linear equation	R^2
Total flavonoids	Rutin	y = 0.0016x + 0.0515	0.998
Total polyphenols	Gallic acid	y = 0.0113x + 0.0783	0.999
Soluble sugar	Glucose	y = 3.7001x + 0.284	0.997
Free amino acid	Glycine	y = 2.732x + 0.115	0.999
Soluble protein	Bovine Serum Albumin	y = 0.204x + 0.284	0.998

Note: x, amount of each analyzed reference standard (mg/mL); y, optical density.

Table S2 Linear equations and correlation coefficients (R^2) of rutin, quercetin and kaempferol.

Compound	Linear equation	R^2
Rutin	y = 43305.6x - 7646	0.998
Quercetin	y = 27489.2x - 7417.2	0.999
Kaempferol	y = 23661.0x - 3687.6	0.998

Note: x, amount of each analyzed injection compound (μg/mL); y, peak area.

Table S3 IC_{50} values of toon-leaf tea extracts for $ABTS\cdot^{+}$ and DPPH· radical scavenging activities

Sample	$ABTS\cdot^{+}$ radical scavenging (mg/mL)	DPPH· radical scavenging (mg/mL)
TTDL	2.32 ± 0.04^{b}	1.78 ± 0.04^{b}
TTLT	1.98 ± 0.03^{a}	1.39 ± 0.02^{a}
TMDL	2.99 ± 0.05^{d}	2.91 ± 0.08^{c}
TMLT	2.67 ± 0.04^{c}	1.64 ± 0.04^{b}

Note: Different letters in the values denote statistically significant differences between groups ($p < 0.05$).

Table S4 Antioxidant activities of toon-leaf tea extracts determined by DPPH assays.

Sample	DPPH· radical scavenging (μmol TE/g DW)
TTDL	149.32 ± 8.24[b]
TTLT	188.33 ± 5.09[c]
TMDL	101.35 ± 6.02[a]
TMLT	154.10 ± 7.91[b]

Note: Different letters in the values denote statistically significant differences between groups ($p < 0.05$).

Figure S1

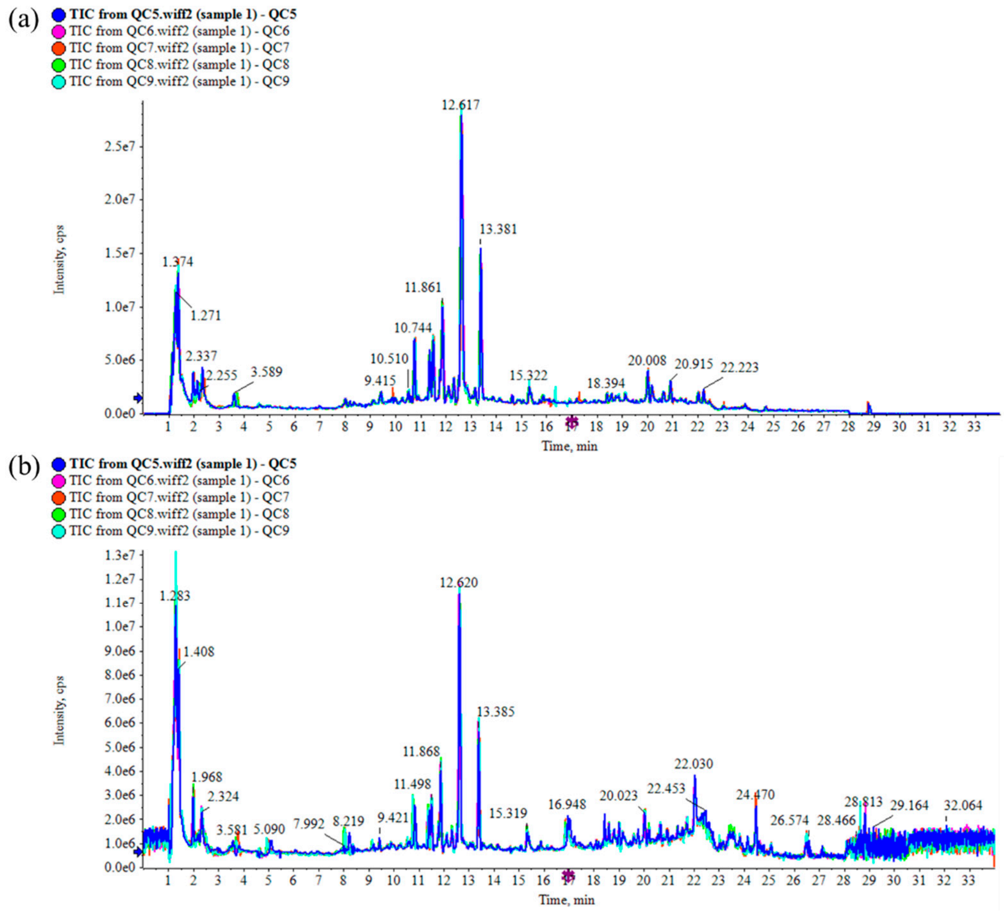

Figure S1. TIC overlay chromatograms of QC samples in positive and negative ion modes. (A) Negative ion mode; (B) Positive ion mode.
